# 662 Developing a Burn Center Process Improvement Program

**DOI:** 10.1093/jbcr/iraf019.291

**Published:** 2025-04-01

**Authors:** Denise Mazzacano Searles, Mark Thomas, Niknam Eshraghi

**Affiliations:** Legacy Oregon Burn Center; Legacy Oregon Burn Center; Legacy Oregon Burn Center

## Abstract

**Introduction:**

Prior to 2018, our burn program lacked a process to assess opportunities for improvement. Mortalities and sentinel events were reviewed at the hospital level, but we lacked a structure for internal multidisciplinary review. Other complications in patient care (infections, graft failure, etc.) were also not addressed systematically. As part of our ABA verification, we implemented a burn unit-specific PI process to address this gap.

**Methods:**

Our Pi program is modeled on the Society of Trauma Nurses’ Trauma Outcomes & Performance Improvement Course, with four levels of review. An experienced burn RN was hired as PI coordinator, working closely with unit management, the program medical director, and other disciplines. At each review level, the team looks for gaps in care, opportunities for improvement, and potential action steps.

**Review levels:**

• Primary – burn coordinator reviews all cases (reviews charts, attends walking rounds, participates in weekly departmental rounds), tracking variances based on agreed-upon triggers for review. Many complications do not advance further

• Secondary – burn coordinator and medical director meet weekly to discuss significant variances and select cases for further review

• Tertiary – burn coordinator, attending physicians, program management, and outreach coordinator meet monthly to review selected variances and ongoing PI work, looking for opportunities for improvement and selecting cases for broader multidisciplinary review

• Quaternary – multidisciplinary review, including burn unit attendings and representatives from several disciplines; this group meets monthly to review complications and mortalities, seeking opportunities for improvement and determining next steps

**Results:**

In 2023, 192 patients and 488 events underwent primary review, 39 cases underwent tertiary review, and 35 cases underwent quaternary review. This process identified multiple opportunities for improvement, including

• Collaboration with PICU colleagues to develop pediatric pain and agitation treatment guidelines

• Standardization of airway cart contents for emergent intubations

• Developing guidelines for silver-impregnated central line dressings to prevent CLABSI

In addition to improved patient outcomes, staff also benefit from this ongoing process through

• Identification of knowledge gaps

• Development of patient care guidelines

• Attending multidisciplinary review to gain deeper insight into complex cases

**Conclusions:**

Our burn unit has developed a robust process improvement program. Gaps in staff education are addressed and practice modifications have been implemented. This PI program benefits both patients and staff, leading to better patient outcomes.

**Applicability of Research to Practice:**

Multiple opportunities for improvement have been identified through this PI process, leading to improved patient care and outcomes, as well as increased staff participation.

**Funding for the Study:**

N/A

